# Glutaminolysis is involved in the activation of mTORC1 in in vitro‐produced porcine embryos

**DOI:** 10.1002/mrd.23516

**Published:** 2021-06-01

**Authors:** Paula R. Chen, Caroline G. Lucas, Lee D. Spate, Randall S. Prather

**Affiliations:** ^1^ Division of Animal Sciences University of Missouri Columbia Missouri USA

**Keywords:** glutamine, mechanistic target of rapamycin, porcine, preimplantation embryo

## Abstract

Glutamine supplementation to porcine embryo culture medium improves development, increases leucine consumption, and enhances mitochondrial activity. In cancer cells, glutamine has been implicated in the activation of mechanistic target of rapamycin complex 1 (mTORC1) to support rapid proliferation. The objective of this study was to determine if glutamine metabolism, known as glutaminolysis, was involved in mTORC1 activation in porcine embryos. Culture with 3.75 mM GlutaMAX improved development to the blastocyst stage compared to culture with 1 mM GlutaMAX, and culture with 0 mM GlutaMAX decreased development compared to all groups with GlutaMAX. Ratios of phosphorylated to total MTOR were increased when embryos were cultured with 3.75 or 10 mM GlutaMAX, which was enhanced by the absence of leucine, but ratios for RPS6K were unchanged. As another indicator of mTORC1 activation, colocalization of MTOR and a lysosomal marker was increased in embryos cultured with 3.75 or 10 mM GlutaMAX in the absence of leucine. Culturing embryos with glutaminase inhibitors decreased development and the ratio of phosphorylated to total MTOR, indicating reduced activation of the complex. Therefore, glutaminolysis is involved in the activation of mTORC1 in porcine embryos, but further studies are needed to characterize downstream effects on development.

## 1 INTRODUCTION

Glutamine is a versatile amino acid with several roles in porcine preimplantation embryo development. Petters et al. (1990) demonstrated that porcine one‐ and two‐cell stage embryos could be cultured to the blastocyst stage in medium containing glutamine without glucose, pyruvate, or lactate, indicating that glutamine acts as an energy source to drive proliferation. As further support, glutamine consumption for the tricarboxylic acid cycle was shown to be consistent from the two‐cell to blastocyst stages in in vitro‐produced porcine embryos (Swain et al., 2002). Moreover, the presence of glutamine in porcine embryo culture medium reduced intracellular H_2_O_2_ concentrations and DNA damage in Day 3 embryos, indicating a role of glutamine in redox regulation (Suzuki et al., 2007). Deep sequencing revealed that several transcripts related to glutamine transport and metabolism were upregulated in in vitro‐produced porcine embryos compared to in vivo counterparts, potentially indicating a deficiency in the medium (Bauer et al., 2010). Following replacement of 1 mM glutamine with 3.75 mM GlutaMAX (alanyl‐*l*‐glutamine) in the porcine embryo culture medium, known as MU3 medium, embryos had enhanced development to the blastocyst stage, normalized glutamine‐related transcript abundance, improved mitochondrial function, and increased leucine consumption (Chen et al., 2018).

In many common cancers, mechanistic target of rapamycin complex 1 (mTORC1) activation and signaling are increased, driving metabolic changes needed to stimulate anabolic processes within the cell (Menon & Manning, 2008). The mTORC1 responds to numerous signals regarding the energy status of the cell to promote growth and proliferation through phosphorylation of downstream targets. The two most highly characterized targets of mTORC1 are ribosomal protein S6 kinase (RPS6K) and eukaryotic translation initiation factor 4E‐binding protein 1 (EIF4EBP1). Activation of RPS6K leads to phosphorylation of targets that enhance translation initiation and elongation, such as ribosomal protein S6 (RPS6) and eukaryotic initiation factor 4B, and phosphorylation of target transcription factors for cell proliferation and survival (Buck et al., 2001; Shahbazian et al., 2006). EIF4EBP1 inhibits translation by binding eukaryotic initiation factor 4E (EIF4E) to prevent cap‐dependent translation; however, phosphorylation of EIF4EBP1 by mTORC1 disrupts this binding, allowing translation to proceed (Yanagiya et al., 2012). Amino acids have been extensively studied for their roles in mTORC1 activation, leading to translocation of the complex to the lysosome and subsequent phosphorylation. For example, glutaminolysis, which is the conversion of glutamine to glutamate through glutaminase (GLS) and to α‐ketoglutarate through glutamate dehydrogenase (GLUD1), has been shown to be involved in mTORC1 activation. Leucine promotes glutaminolysis by allosterically binding and activating GLUD1, indicating an important interaction between these two amino acids (Couée & Tipton, 1989). Treatment of U2OS human osteosarcoma cells with glutaminolysis inhibitors decreased phosphorylation of mTORC1 targets, RPS6K, and RPS6 (Durán et al., 2012). On the other hand, supplementation of α‐ketoglutarate in the medium of amino acid‐starved U2OS cells restored phosphorylation of mTORC1 targets and colocalization with the lysosome.

Cancer cells and blastomeres of preimplantation embryos are known to have metabolic similarities (Redel et al., 2012), and connections between mTORC1 and early embryonic development have also been established. For instance, mouse blastocyst‐stage embryos have been shown to have increased phosphorylation of RPS6K and EIF4EBP1 compared to previous developmental stages (Zamfirescu et al., 2020). Culture of blastocyst‐stage embryos without amino acids for 5 h decreased phosphorylation of mTORC1 targets, confirming the importance of these molecules in mTORC1 activation (Zamfirescu et al., 2020). Additionally, TE of rabbit blastocyst‐stage embryos was shown to have increased phosphorylation of mTORC1 targets compared to the inner cell mass (ICM) (Gürke et al., 2016). This is in agreement with the fact that the TE is more metabolically active as opposed to the ICM, as indicated by increased oxygen consumption, amino acid turnover, and ATP production (Houghton, 2006). In porcine TE cultures, addition of leucine (0.4 mM) or glutamine (2 mM) to the medium after serum starvation and amino acid depletion increased proliferation and phosphorylation of mTORC1 targets in a temporal manner (Kim et al., 2013). However, effects of amino acids in the culture medium on mTORC1 activation have not been investigated in porcine preimplantation embryos thus far.

As increased glutamine in the culture medium was shown to improve development to the blastocyst stage, the objective of this study was to determine if glutaminolysis is involved in the activation of mTORC1 and its targets in porcine preimplantation embryos. First, GlutaMAX was supplemented into the porcine embryo culture medium with or without leucine to investigate its role in mTORC1 activation and interactions between these two amino acids. Then, embryos were cultured with glutaminolysis inhibitors to understand if blocking glutamine metabolism decreased mTORC1 activation.

## 2 RESULTS

### 2.1 GlutaMAX supplementation improves development in the presence or absence of leucine

**Figure 1:**
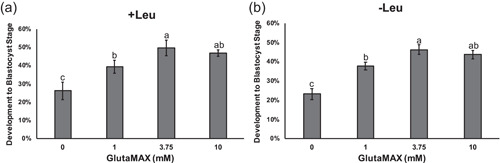
Development to the blastocyst‐stage on Day 6 for embryos cultured in different concentrations of GlutaMAX with (a) or without (b) leucine. Data presented as means ± *SEM* across six replicates (*n* = 600 presumptive zygotes per concentration). Different letters (^a,b,c^) indicate statistical differences (*p* < 0.05)

A concentration curve of glutamine in the form of GlutaMAX (0, 1, 3.75, and 10 mM) was used to assess the effects of this amino acid on development in the presence or absence of leucine. MU3 contains 3.75 mM GlutaMAX and 0.2 mM leucine (Chen et al., 2018), and removal of leucine does not impact development to the blastocyst stage nor total number of nuclei in the embryos (Figure S1a,b). However, 1.8 mM leucine in the medium decreased development to the blastocyst stage compared with 0 or 0.2 mM (25.4 ± 6.1% vs. 45.5 ± 3.0% or 45.9 ± 3.8%, respectively). When leucine was present in the medium, 0 mM GlutaMAX decreased development to the blastocyst stage compared to all other groups, and 3.75 mM GlutaMAX increased development compared to 1 mM GlutaMAX (49.7 ± 4.2% vs. 39.6 ± 3.6%; Figure 1a). Similarly, when embryos were cultured without leucine, 0 mM GlutaMAX decreased development to the blastocyst stage compared to the other groups, and 3.75 mM GlutaMAX increased development compared to 1 mM GlutaMAX (46.2 ± 2.6% vs. 37.8 ± 2.1%; Figure 1b).

### 2.2 Phosphorylation of MTOR and RPS6K is dependent upon the concentration of GlutaMAX

**Figure 2:**
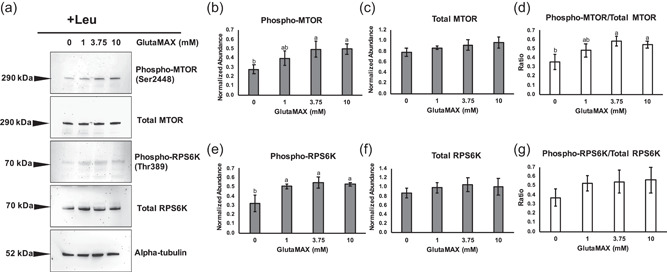
Western blot analysis of phosphorylated MTOR (Ser2448), total MTOR, phosphorylated ribosomal protein S6 kinase (RPS6K) (Thr389), and total RPS6K for blastocyst‐stage embryos cultured with leucine. (a) Representative blots for embryos cultured with a dose curve of GlutaMAX. Target protein molecular weights in kilodaltons (kDa) are depicted to the left of each blot. Alpha‐tubulin was used as the loading control. Densitometry analysis across three replicates (*n* = 100 embryos per concentration) for (b) phosphorylated MTOR, (c) total MTOR, (d) ratio of phosphorylated to total MTOR, (e) phosphorylated RPS6K, (f) total RPS6K, and (g) ratio of phosphorylated to total RPS6K. Different letters (^a,b^) indicate statistical differences (*p* < 0.05). Absence of superscripts above bars indicates that statistical differences were not observed between any of the groups (*p* > 0.05)

To determine if glutamine and leucine regulated mTORC1 activation in porcine embryos, abundances of total and phosphorylated MTOR and RPS6K, a canonical target of mTORC1, were measured. When leucine was present, abundance of total MTOR was not observed to be different between any groups; however, abundance of phosphorylated MTOR was increased in embryos cultured with 3.75 mM or 10 mM GlutaMAX compared with 0 mM GlutaMAX (Figure 2a–c). Moreover, the ratio of phosphorylated to total MTOR was increased in the 3.75 and 10 mM groups compared with the 0 mM group (Figure 2d). A difference in abundance of total RPS6K was not detected between groups, but abundance of phosphorylated RPS6K was increased in embryos cultured with 1, 3.75, or 10 mM GlutaMAX compared to 0 mM (Figures 2a,e, and f). However, the ratio of phosphorylated to total RPS6K was not different between groups (Figure 2g).

**Figure 3:**
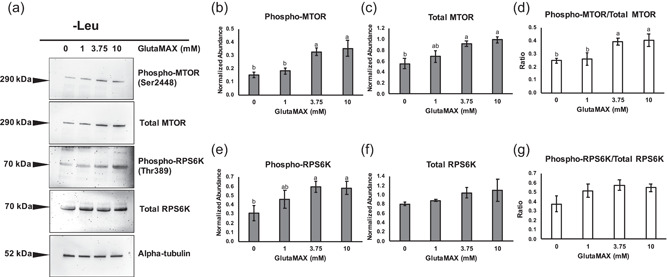
Western blot analysis of phosphorylated MTOR (Ser2448), total MTOR, phosphorylated RPS6K (Thr389), and total RPS6K for blastocyst‐stage embryos cultured without leucine. (a) Representative blots for embryos cultured with a dose curve of GlutaMAX. Target protein molecular weights in kilodaltons (kDa) are depicted to the left of each blot. Alpha‐tubulin was used as the loading control. Densitometry analysis across three replicates (*n* = 100 embryos per concentration) for (b) phosphorylated MTOR, (c) total MTOR, (d) ratio of phosphorylated to total MTOR, (e) phosphorylated RPS6K, (f) total RPS6K, and (g) ratio of phosphorylated to total RPS6K. Different letters (^a,b^) indicate statistical differences (*p* < 0.05). Absence of superscripts above bars indicates that statistical differences were not observed between any of the groups (*p* > 0.05). RPS6K, ribosomal protein S6 kinase

When leucine was absent, abundance of total MTOR was increased in the 3.75 and 10 mM groups compared to embryos cultured with 0 mM GlutaMAX, and abundance of phosphorylated MTOR was increased in embryos cultured with 3.75 or 10 mM compared to those cultured in 0 or 1 mM GlutaMAX (Figure 3a–c). The ratio of phosphorylated to total MTOR was increased in the 3.75 and 10 mM groups compared to the 0 and 1 mM groups (Figure 3d). Abundance of total RPS6K was not observed to be different between any groups; however, abundance of phosphorylated RPS6K was increased in the 3.75 and 10 mM groups compared to 0 mM (Figures 3a,e, and f). Differences in the ratio of phosphorylated to total RPS6K was not detected between groups (Figure 3g).

### 2.3 GlutaMAX increases colocalization of MTOR with lysosomes in the absence of leucine

**Figure 4:**
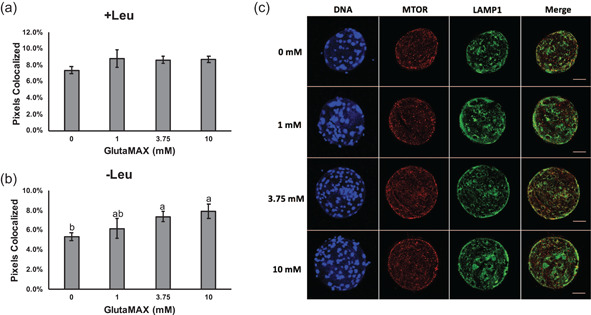
Colocalization of MTOR and LAMP1 in porcine blastocyst‐stage embryos as an indicator of mTORC1 activation. Percentage of MTOR and LAMP1 pixels colocalized for embryos cultured with (a) leucine and without (b) leucine. Data presented as mean ± *SEM* across three replicates (*n* = 15 embryos per concentration). Different letters (^a,b^) indicate statistical differences (*p* < 0.05). Absence of superscripts above bars indicates that statistical differences were not observed between any of the groups (*p* > 0.05). (c) Representative images of embryos cultured in different concentrations of GlutaMAX without leucine and stained for DNA (Hoechst 33342), MTOR, and LAMP1. Scale bars = 50 μm. RPS6K, ribosomal protein S6 kinase

Colocalization of MTOR and lysosomal‐associated membrane protein 1 (LAMP1) was evaluated by using confocal microscopy in porcine blastocyst‐stage embryos as mTORC1 is activated at the lysosomal membrane. When leucine was present in the medium, there were no detectable differences in colocalization between any groups (Figure 4a). When leucine was absent, MTOR and LAMP1 colocalization was increased in the 3.75 mM and 10 mM groups compared to culture with 0 mM GlutaMAX (7.4 ± 0.5% and 8.0 ± 0.7%, respectively, vs. 5.4 ± 0.4%; Figure 4b,c), indicating that glutamine‐sufficient conditions may act as another signal for translocation of mTORC1 to the lysosome.

### 2.4 Pharmacological inhibition of GLS decreases development of porcine embryos

**Figure 5:**
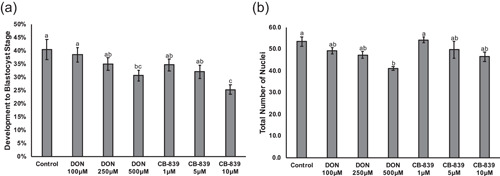
Developmental parameters for embryos treated with glutaminase (GLS) inhibitors. (a) Percentage of embryos developing to the blastocyst stage on Day 6 after culture in MU3 (control), MU3 with 100, 250, or 500 µM DON, and MU3 with 1, 5, or 10 µM CB‐839. Values determined across five replicates (*n* = 250 presumptive zygotes per treatment). (b) Total number of nuclei in Day 6 blastocyst‐stage embryos. Values determined across five replicates (*n* = 60‐75 embryos per treatment). Data presented as mean ± *SEM*. Different letters (^a,b,c^) indicate statistical differences (*p* < 0.05). DON, 6‐diazo‐5‐oxo‐l‐norleucine; RPS6K, ribosomal protein S6 kinase

Activity of GLS, the rate‐limiting enzyme of glutaminolysis, was targeted by using dose curves of two different compounds. 6‐Diazo‐5‐oxo‐l‐norleucine (DON) is a commonly used glutamine antagonist (Durán et al., 2012) and enters the catalytic center of GLS but also inhibits activity of other enzymes. N‐[5‐[4‐[6‐[[2‐[3‐(trifluoromethoxy)phenyl]acetyl]amino]‐3‐pyridazinyl]butyl]‐1,3,4‐thiadiazol‐2‐yl]‐2‐pyridineacetamide (CB‐839) allosterically inhibits GLS and has demonstrated higher specificity than other inhibitors. The highest concentrations of DON (500 μM) or CB‐839 (10 μM) decreased development to the blastocyst stage compared to control embryos cultured in MU3 (30.6 ± 2.0% or 25.3 ± 1.8%, respectively, vs. 40.5 ± 3.8%; Figure 5a). Moreover, embryos cultured with 500 μM DON had decreased total cell numbers in the blastocyst‐stage embryos compared to the control (41.1 ± 0.8 vs. 53.4 ± 2.2), but there was no detectable difference in cell numbers between embryos cultured with 10 μM CB‐839 and the control embryos (Figure 5b).

### 2.5 Treatment with GLS inhibitors disrupts activation of mTORC1 in porcine embryos

**Figure 6:**
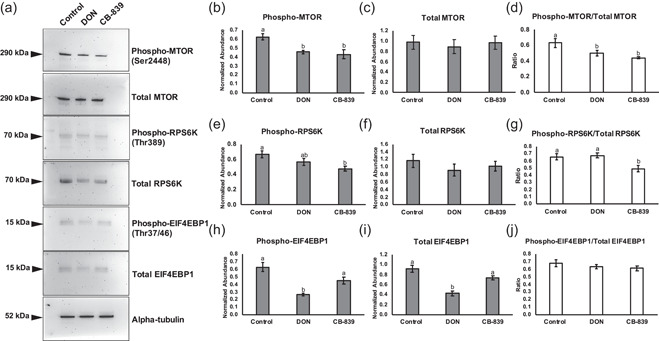
Western blot analysis of phosphorylated MTOR (Ser2448), total MTOR, phosphorylated RPS6K (Thr389), total RPS6K, phosphorylated EIF4EBP1 (Thr37/46), and total EIF4EBP1 for blastocyst‐stage embryos cultured GLS inhibitors. (a) Representative blots for embryos cultured in MU3 (control), MU3 with 500 µM DON, or MU3 with 10 µM CB‐839. Target protein molecular weights in kilodaltons (kDa) are depicted to the left of each blot. Alpha‐tubulin was used as the loading control. Densitometry analysis across three replicates (*n* = 100 embryos per treatment) for (b) phosphorylated MTOR, (c) total MTOR, (d) ratio of phosphorylated to total MTOR, (e) phosphorylated RPS6K, (f) total RPS6K, (g) ratio of phosphorylated to total RPS6K, (h) phosphorylated EIF4EBP1, (i) total EIF4EBP1, and (j) ratio of phosphorylated to total EIF4EBP1. Different letters (^a,b^) indicate statistical differences (*p* < 0.05). Absence of superscripts above bars indicates that statistical differences were not observed between any of the groups (*p* > 0.05). DON, 6‐diazo‐5‐oxo‐l‐norleucine; GLS, glutaminase; RPS6K, ribosomal protein S6 kinase

The highest concentrations of DON and CB‐839 were selected to determine the impact of GLS inhibition on activation of mTORC1. Abundance of total MTOR was not observed to be different when embryos were cultured with DON or CB‐839, but abundance of phosphorylated MTOR was decreased with treatment of either inhibitor compared to the control (Figure 6a–c). Additionally, the ratio of phosphorylated to total MTOR was decreased in DON and CB‐839‐cultured embryos compared to the control (Figure 6d). Differences in abundance of total RPS6K were not detected between groups, but abundance of phosphorylated RPS6K was decreased when embryos were cultured with CB‐839 compared to the control (Figures 6a,e, and f). Additionally, the ratio of phosphorylated to total RPS6K was decreased in CB‐839‐cultured embryos compared to the control or those cultured with DON (Figure 6g). In this portion of the study, abundance of EIF4EBP1 was measured. Interestingly, total and phosphorylated EIF4EBP1 was decreased when embryos were cultured with DON compared to the control and those cultured with CB‐839 (Figures 6a,h, and i), but ratios were not detected to be different between any group (Figure 6j).

## 3 DISCUSSION

Several signals are sensed by the cell resulting in phosphorylation and activation of mTORC1 to promote anabolic processes, such as translation, lipogenesis, and DNA synthesis, through phosphorylation of downstream targets (Laplante & Sabatini, 2012; Porstmann et al., 2008; Ye et al., 2014). Over the past decade, amino acids have gained considerable interest in the regulation of mTORC1. Specifically, the metabolism of glutamine, known as glutaminolysis, has been shown to in involved in the activation of mTORC1 in somatic and cancer cells (Durán et al., 2012; Jewell et al., 2015). However, little is known about amino acid‐dependent regulation of mTORC1 in preimplantation embryos. Previously, we observed that replacement of 1 mM glutamine with 3.75 mM GlutaMAX, an alanyl‐*l*‐glutamine dipeptide, in our porcine embryo culture medium improved development to the blastocyst stage, normalized abundance of glutamine‐related transcripts, and increased mitochondrial activity (Chen et al., 2018). Moreover, embryos cultured with 3.75 mM GlutaMAX consumed more leucine from the culture medium than control embryos cultured with 1 mM glutamine (Chen et al., 2018). Thus, the aim of the current study was to determine if glutamine in porcine embryo culture medium contributed to mTORC1 activation in the presence or absence of leucine, another well‐known activator of mTORC1.

In the current study, a concentration curve of glutamine in the form of GlutaMAX (0, 1, 3.75, or 10 mM) was used to determine its effects on development to the blastocyst stage and subsequent activation of mTORC1. Absence (0 mM) of GlutaMAX from the medium decreased development to the blastocyst stage compared to any group with GlutaMAX, which is similar to observations with *l*‐glutamine (Chen et al., 2018; Suzuki et al., 2007). This indicates that glutamine, as a free amino acid or in a dipeptide (GlutaMAX), has beneficial effects on development of porcine embryos in vitro. Notably, development to the blastocyst stage was increased when 3.75 mM GlutaMAX was added to the culture medium with or without leucine compared to 1 mM GlutaMAX, confirming concentration‐dependent responses to glutamine during progression from cleavage to blastocyst stages in porcine embryos. Concentration‐dependent responses for other amino acids, such as arginine and glycine, have also been observed during in vitro culture of bovine and porcine embryos (Herrick et al., 2016; Redel et al., 2016a; Redel et al., 2016b).

To determine if the concentration of GlutaMAX in the medium impacted activation of mTORC1 in the presence or absence of leucine, blastocyst‐stage embryos were collected for each concentration, and phosphorylation of MTOR and RPS6K, and colocalization of MTOR and LAMP1 were assessed. In the presence of leucine, abundance of phosphorylated MTOR and the ratio of phosphorylated to total MTOR were increased in embryos cultured with 3.75 or 10 mM GlutaMAX compared to embryos cultured with 0 mM GlutaMAX, thus illustrating a concentration‐dependent response to glutamine on activation of mTORC1. A similar pattern in MTOR phosphorylation was observed when embryos were cultured without leucine; however, embryos cultured with 0 and 1 mM GlutaMAX showed decreased abundance of phosphorylated MTOR and ratio of phosphorylated to total MTOR compared to the 3.75 and 10 mM groups. This is congruent with the observation that embryos cultured with 3.75 mM GlutaMAX had increased consumption of leucine from the medium compared to those cultured with 1 mM *l*‐glutamine (Chen et al., 2018). A pool of intracellular glutamine in cells is required for the solute carrier family 7 member 5 (SLC7A5) and solute carrier family 3 member 2 (SLC3A2) bidirectional transporters to pump glutamine out of the cell and promote leucine influx into the cell. Transfection of small interfering RNAs targeting *SLC7A5* or *SLC3A2* in HeLa cells decreased activation of mTORC1 and subsequently resulted in reduced cell size (Nicklin et al., 2009). Interestingly, abundance of total MTOR was decreased in embryos cultured with 0 mM GlutaMAX compared to those cultured with 3.75 or 10 mM GlutaMAX in the absence of leucine. Thus, the absence of both leucine and glutamine from the culture medium may promote turnover of MTOR, which has not been reported previously. Phosphorylation of RPS6K has been widely used as an indicator of mTORC1 activation in response to amino acids (Durán et al., 2012; Kim et al., 2013; Laplante & Sabatini, 2012). When embryos were cultured with 0 mM GlutaMAX, phosphorylated RPS6K was decreased compared to embryos cultured with GlutaMAX, but the ratio of phosphorylated to total RPS6K was not different. Phosphorylation of RPS6K by mTORC1 has been shown to stabilize the protein and prevent proteasomal degradation (Zhang et al., 2014); however, no difference in abundance of total RPS6K was detected between groups. Translocation of mTORC1 to the lysosome is a prerequisite for activation of the complex; thus, colocalization of MTOR and lysosomal markers has been used as an indicator of mTORC1 activation (Durán et al., 2012; Frias et al., 2020). When leucine was absent from the culture medium, embryos cultured with 3.75 or 10 mM GlutaMAX had increased colocalization of MTOR and a lysosomal membrane component, LAMP1, compared to embryos cultured with 0 mM GlutaMAX. However, differences in colocalization were not observed when leucine was present. Thus, leucine potentially produces a stronger signal for mTORC1 translocation, but glutamine may compensate when leucine is absent.

To further clarify the role of glutaminolysis in the activation of mTORC1, embryos were cultured with inhibitors of GLS, which converts glutamine to glutamate. DON is a glutamine antagonist that has been shown to inhibit the activity of GLS, but it also has other enzymatic targets, such as those involved in nucleotide and asparagine synthesis (Ahluwalia et al., 1990; Lemberg et al., 2018; Rosenbluth et al., 1976). CB‐839 is a highly selective inhibitor of GLS and is currently in clinical trials for treating several cancer types in combination with other therapeutics (Jin et al., 2020; Zhao et al., 2020). In the current study, culture with DON or CB‐839 decreased development to the blastocyst stage, and culture with DON decreased total cell number in the blastocyst‐stage embryos. DON exposure during embryogenesis has been shown to induce limb malformations in mice and interferes with purine metabolism (Greene & Kochhar, 1975), which may explain the decreased cell number in the DON‐treated porcine embryos. DON and another GLS inhibitor, bis‐2‐(5‐phenylacetamido‐1,3,4‐thiadiazol‐2‐yl)ethyl sulfide (BPTES), have been shown to decrease α‐ketoglutarate production and phosphorylation of RPS6K in U2OS cells (Durán et al., 2012). Regarding the current study, culture with DON or CB‐839 decreased the abundance of phosphorylated MTOR and the ratio of phosphorylated to total MTOR, but only CB‐839 decreased phosphorylated RPS6K and the ratio of phosphorylated to total RPS6K in porcine embryos. The observed discrepancies in effects of the inhibitors may be due to differences in their range of targets. However, culturing porcine embryos with CB‐839, which is more specific for GLS, confirmed that interfering with glutaminolysis does impair activation of mTORC1. Additionally, in this portion of the study, we were able to detect another target of mTORC1, phosphorylated and total EIF4EBP1 in the porcine embryo lysate (Laplante & Sabatini, 2012). Interestingly, culture with DON decreased abundance of total EIF4EBP1, likely resulting in decreased phosphorylated EIF4EBP1 compared to the other groups. The exact mechanism by which DON decreases EIF4EBP1 is not known, but it may be a consequence of the lack of specificity of DON (Lemberg et al., 2018).

The results in the present study build upon previous studies highlighting the importance of glutamine in culture of preimplantation embryos by demonstrating that this amino acid is involved in the regulation of mTORC1, a key growth‐promoting complex. The concentration‐dependent effects of glutamine on development to the blastocyst stage were mirrored by indicators of mTORC1 activation, which were more prominent in the absence of leucine. Moreover, inhibition of glutaminolysis during culture decreased development to the blastocyst stage and activation of mTORC1, revealing that this metabolic process promotes viability of the embryos. Further studies are needed to characterize the effects of mTORC1 activation in response to amino acids on subsequent development and dysregulation of the complex by in vitro culture.

## 4 MATERIALS AND METHODS

### 4.1 Chemical components

All chemicals were purchased from Sigma Chemical Company unless stated otherwise.

### 4.2 Ethics statement

Collection of ovaries from prepubertal gilts was in accordance with approved protocol and standard operating procedures by the Animal Care and Use Committee of the University of Missouri.

### 4.3 In vitro production of embryos

Ovaries from prepubertal gilts were collected at the Smithfield Foods, Inc. abattoir in Milan, MO. Follicles (3–6 mm in diameter) were aspirated by using an 18‐gauge needle attached to a 10‐ml syringe. Cumulus‐oocyte complexes with at least three layers of cumulus cells were matured in FLI‐containing medium as described previously (Yuan et al., 2017). Cumulus cells of matured oocytes were removed by gentle vortexing for 2 min in 0.1% (wt/vol) hyaluronidase in Tyrode's Lactate 4‐(2‐hydroxyethyl)‐1‐piperazineethanesulfonic acid (TL‐HEPES)‐buffered saline with 0.1% PVA. Oocytes at the metaphase II (MII) stage demonstrating extrusion of the first polar body were selected for in vitro fertilization (IVF). MII oocytes were placed into 50 μl droplets of IVF medium (modified Tris‐buffered medium containing 2 mg/ml fatty acid‐free bovine serum albumin and 2 mM caffeine) in a mineral oil overlay and maintained at 38.5°C until sperm were added. All experiments used sperm obtained from two domestic boars, and sperm preparation and addition were described by Redel et al. (2012).

For the experiments involving the concentration curve of glutamine in the form of an alanyl‐*l*‐glutamine dipeptide, GlutaMAX, presumptively fertilized oocytes were removed, washed, and transferred in groups of 50 into four‐well dishes containing 500 µl of MU3 porcine embryo medium with 0, 1, 3.75, or 10 mM GlutaMAX (Chen et al., 2018). Furthermore, embryos were cultured with or without 0.2 mM leucine, and all groups were maintained in an atmosphere of 5% O_2_, 5% CO_2_, and 90% N_2_ at 38.5°C until Day 6 (D6) post‐fertilization. The percentage developed to blastocyst stage on D6 were recorded for each treatment.

For the concentration curve of leucine, fertilized oocytes were cultured in groups of 50 into four‐well dishes containing 500 µl of MU3 porcine embryo medium with 0, 0.2, 0.6, 1.2, or 1.8 mM leucine. The percentage developed to blastocyst stage on D6 were recorded for each treatment. Blastocyst‐stage embryos were fixed in 2% paraformaldehyde for 20 min at room temperature, stained with Hoechst 33342 (10 μg/m) for 10 min, and total number of nuclei was recorded after visualization by using a UV filter attached to a Nikon Eclipse E600 microscope (Nikon).

For the experiments involving the GLS inhibitors, fertilized oocytes were removed, washed, and transferred in groups of 50 into four‐well dishes containing 500 µl of MU3 porcine embryo medium with 0, 100, 250, or 500 μM DON or 0, 1, 5, or 10 μM N‐[5‐[4‐[6‐[[2‐[3‐(trifluoromethoxy)phenyl]acetyl]amino]‐3‐pyridazinyl]butyl]‐1,3,4‐thiadiazol‐2‐yl]‐2‐pyridineacetamide (CB‐839). All groups were maintained in an atmosphere of 5% O_2_, 5% CO_2_, and 90% N_2_ at 38.5°C until D6 post‐fertilization. The percentage developed to blastocyst stage on D6 and total number of nuclei were recorded for each treatment.

### 4.4 Western blotting

Pools of D6 blastocyst‐stage embryos (*n* = 100 per treatment per replicate) were washed in TL‐HEPES, snap frozen, and stored at −80°C. Embryos were lysed in 2X Laemmli buffer (Bio‐Rad Laboratories) with 5% β‐mercaptoethanol, and proteins were separated on a 4%–20% sodium dodecyl sulfate‐polyacrylamide gel electrophoresis gel. Afterwards, proteins were dry transferred to polyvinylidene difluoride membranes by using an iBlot Dry Transfer System (Thermo Fisher Scientific). Membranes were blocked with 5% nonfat dry milk in tris‐buffered saline with 0.1% Tween 20 and incubated with primary antibodies overnight at 4°C for anti‐phosphorylated MTOR (Ser2448; 1:1000; 5536; Cell Signaling Technology), anti‐phosphorylated RPS6K (Thr389; 1:500; 9206; Cell Signaling Technology), or anti‐phosphorylated EIF4EBP1 (Thr37 and Thr46; 1:500; 2855; Cell Signaling Technology). Membranes were washed and incubated with goat anti‐rabbit immunoglobulin G (IgG) (1:5000; 31466; Invitrogen) or rabbit anti‐mouse IgG (1:5000; 31450; Invitrogen) secondary antibodies for 1 h at room temperature. Following washing, Pierce ECL 2 Western blot analysis Substrate (Thermo Fisher Scientific) was applied to the membranes for 3 min, and blots were visualized after a 10 min exposure by using FOTO/Analyst PC Image software (version 10) with the same settings for each replicate. Membranes were stripped for subsequent probing with antibodies for anti‐MTOR (1:1000; 2983; Cell Signaling Technology), anti‐RPS6K (1:1000; 9202; Cell Signaling Technology), anti‐EIF4EBP1 (1:500; 9452; Cell Signaling Technology), or anti‐alpha‐tubulin (1:5000; A01410; GenScript Biotech). Densitometries were evaluated against alpha‐tubulin for normalization by using Fiji (available at: https://imagej.net/Fiji). Whole blot controls for each antibody by using blastocyst‐stage embryos (50–100 embryos per target) are shown in Figures S2. Negative controls incubated with normal rabbit or mouse sera and secondary antibodies followed by anti‐alpha‐tubulin are shown in Figures S3a,b.

### 4.5 Confocal microscopy

Blastocyst‐stage embryos were fixed in 2% paraformaldehyde for 20 min at room temperature. Fixed embryos were permeabilized in 0.1% Triton X‐100 in TL‐HEPES for 1 h at room temperature and blocked in 2% goat serum overnight at 4°C. Embryos were incubated with anti‐MTOR (1:50; 2983; Cell Signaling Technology) and anti‐LAMP1 (1:50; MCA2315GA; Bio‐Rad) antibodies overnight at 4°C. Then, embryos were incubated with goat anti‐rabbit IgG tetramethylrhodamine (1:250; T2769; Thermo Fisher Scientific) and goat anti‐mouse IgG FITC (1:250; STAR120F; Bio‐Rad) secondary antibodies for 1 h at room temperature, stained with Hoechst 33342 (10 μg/ml) for 10 min, and mounted for acquisition of 7–9 μm z‐stacks at 20x on a Leica TCP SP8 STED confocal microscope at the University of Missouri Molecular Cytology Core. The excitation/emission wavelengths were 580/610 nm for red, 490/560 nm for green, and 420/480 nm for 4′,6‐diamidino‐2‐phenylindole. Colocalization of MTOR and LAMP1 in z‐stacks was assessed by using Fiji. Percentage of pixels colocalized was measured within the area of the embryos in each image. A negative control incubated with both secondary antibodies is shown in Figures S4.

### 4.6 Statistical analysis

All experiments were repeated at least three times so that replicate variation could be assessed. Development to the blastocyst stage was analyzed by a generalized linear model (PROC GENMOD). Total number of nuclei, densitometries, and pixels colocalized were analyzed by linear mixed models (PROC MIXED). Treatment was modeled as a fixed factor, and biological replicate was modeled as a random factor. The Shapiro‐Wilk test was used for assessing the normality assumption for each experiment, and data were log transformed if deviation from the normality assumption was detected. Significance was discovered by using least square estimates followed by Tukey's honest significant difference test. Type I error rate was controlled at a level of 0.05. Analyses were conducted by using SAS version 9.4 (SAS Institute).

## CONFLICT OF INTERESTS

The authors declare that there are no conflict of interests.

## AUTHOR CONTRIBUTIONS

Paula R. Chen, Caroline G. Lucas, and Randall S. Prather conceived and designed the experiments for the study. Paula R. Chen, Caroline G. Lucas, and Lee D. Spate conducted the experiments. Paula R. Chen and Caroline G. Lucas analyzed the data. Paula R. Chen wrote the manuscript. All authors revised and accepted the manuscript.

### PEER REVIEW

The peer review history for this article is available at https://publons.com/publon/10.1002/mrd.23516

## Supporting information

Supplementary information.

Supplementary information.

Supplementary information.

Supplementary information.

Supplementary information.

## Data Availability

The data that support the findings of this study are available upon request from the corresponding author.
