# Supplementary figures and images for: Glutaminolysis is involved in the activation of mTORC1 in in vitro‐produced porcine embryos
Source: Mol Reprod Dev. 2021 Jun 1;88(7):490–9. doi: 10.1002/mrd.23516 (PMC8361685; doi:10.1002/mrd.23516)

**A**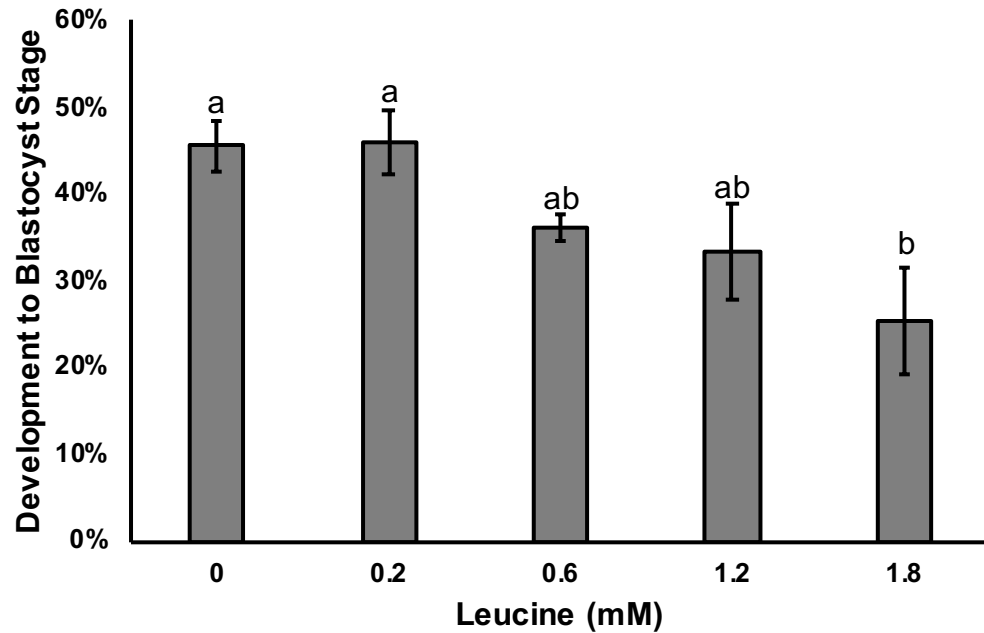**B**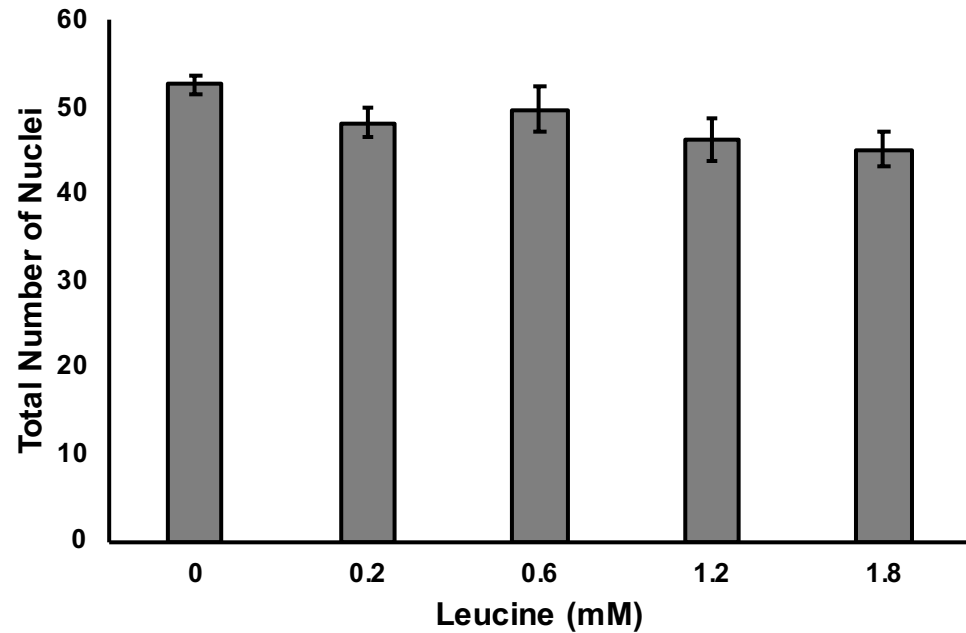

Supplement: Supplementary file 1 — Supplementary information. [file MRD-88-490-s002.pdf]

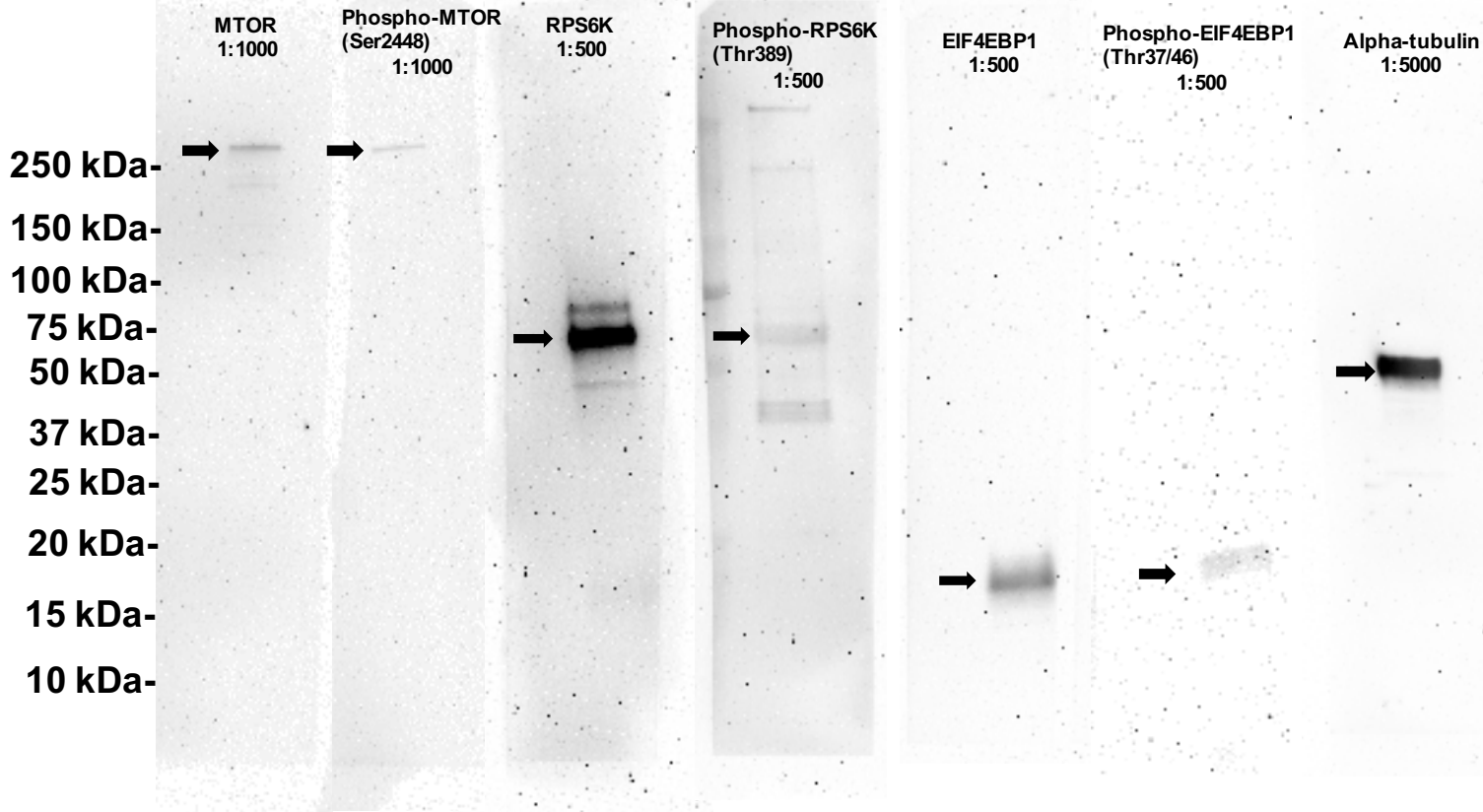

Supplement: Supplementary file 2 — Supplementary information. [file MRD-88-490-s004.pdf]

**A****Anti-rabbit IgG Only**

Gln (mM)    0    1    3.75    10

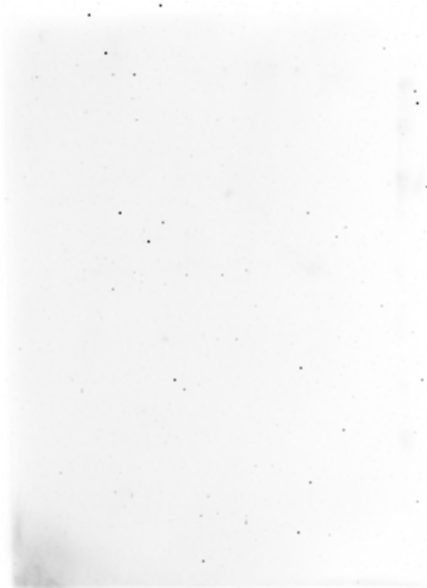**Alpha-tubulin**

0    1    3.75    10

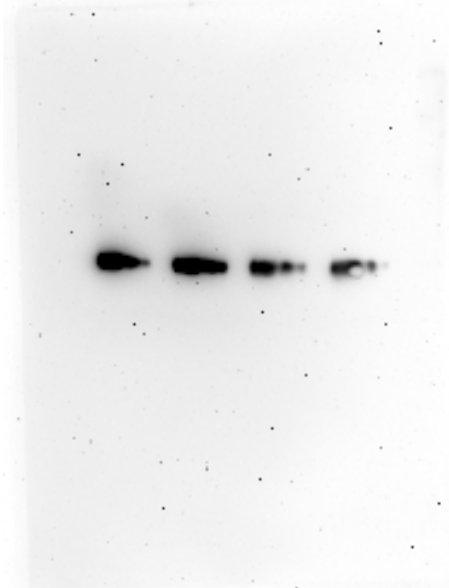**B****Anti-mouse IgG Only**

Gln (mM)    0    1    3.75    10

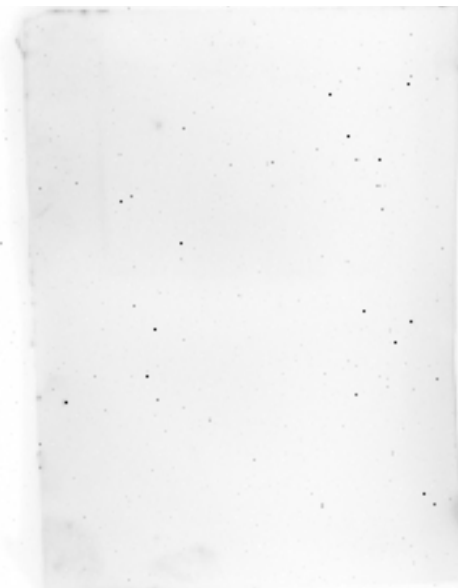**Alpha-tubulin**

0    1    3.75    10

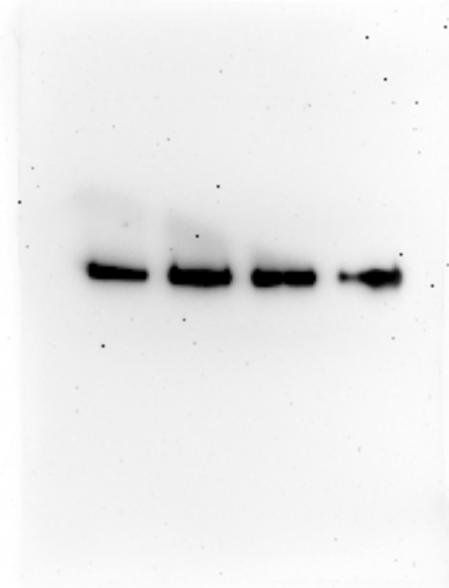

Supplement: Supplementary file 3 — Supplementary information. [file MRD-88-490-s005.pdf]

**DNA**

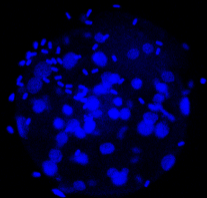

**MTOR**  
(Anti-rabbit  
IgG Only)

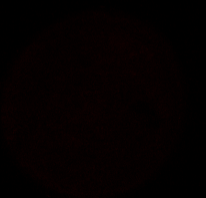

**LAMP1**  
(Anti-mouse  
IgG Only)

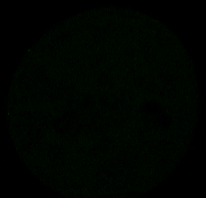

Supplement: Supplementary file 4 — Supplementary information. [file MRD-88-490-s003.pdf]
