# Supplementary material for: Glutaminolysis is involved in the activation of mTORC1 in in vitro‐produced porcine embryos
Source: Mol Reprod Dev. 2021 Jun 1;88(7):490–9. doi: 10.1002/mrd.23516 (PMC8361685; doi:10.1002/mrd.23516)
Supplement: Supplementary file 5 — Supplementary information. [file MRD-88-490-s001.docx]

Supplementary Figure 1. Developmental parameters for embryos cultured in different concentrations of leucine. (A) Percentage of embryos developing to the blastocyst stage on day 6 after culture in 0, 0.2 (control), 0.6, 1.2, or 1.8▒mM leucine. Values determined across 4 replicates (n▒=▒160 presumptive zygotes per treatment). (B) Total number of nuclei in day 6 blastocyst-stage embryos. Values determined across 4 replicates (n▒=▒60 embryos per treatment per replicate). Data presented as means ± SEM. Different letters (a,b) indicate statistical differences (P▒<▒0.05). Absence of superscripts above bars indicates that statistical differences were not observed between any of the groups (P▒>▒0.05).

Supplementary Figure 2. Full western blots for each protein target with molecular weight reference ladder to the left. Protein lysates of 50 blastocyst-stage embryos cultured in MU3 were used for total MTOR and phosphorylated MTOR, respectively. Protein lysates of 100 blastocyst-stage embryos cultured in MU3 were used for total RPS6K, phosphorylated RPS6K, total EIF4EBP1, phosphorylated EIF4EBP1, and alpha-tubulin, respectively. Primary antibody concentrations are shown below target names with respective secondary antibodies diluted 1:5000 except for alpha-tubulin (dilution of 1:20000).

Supplementary Figure 3. Negative control western blots for embryos cultured with different concentrations of GlutaMAX. Representative blots for (A) anti-rabbit IgG and (B) anti-mouse IgG by using protein lysates of 100 blastocyst-stage embryos for each glutamine concentration. Alpha-tubulin was used as the loading control.

Supplementary Figure 4. Representative images of negative controls for confocal microscopy. Negative control was repeated for each replicate of imaging. Blastocyst-stage embryos were incubated with only anti-rabbit IgG and anti-mouse IgG secondary antibodies along with Hoechst 33342 (DNA), and z-stacks were acquired as described.
